# Metformin as an Emerging Pollutant in the Aquatic Environment: Occurrence, Analysis, and Toxicity

**DOI:** 10.3390/toxics12070483

**Published:** 2024-07-02

**Authors:** Yueyue Zheng, Yongjian Shao, Yinan Zhang, Zhiquan Liu, Zirui Zhao, Ranyun Xu, Jiafeng Ding, Wenbing Li, Binhao Wang, Hangjun Zhang

**Affiliations:** 1 College of Life and Environmental Sciences, Hangzhou Normal University, Hangzhou 311121, China; 2 School of Engineering, Hangzhou Normal University, Hangzhou 311121, China; 3 State Environmental Protection Key Laboratory of Environmental Health Impact Assessment of Emerging Contaminants, Shanghai Academy of Environment Sciences, Shanghai 200233, China

**Keywords:** metformin, aquatic environment, occurrence, analysis, toxicity

## Abstract

The use of human and veterinary drugs has led to the accumulation of pharmaceuticals in various aquatic environments at progressively increasing levels, exhibiting strong ecological risks. Metformin is widely used as a first-line prescription drug for the treatment of type 2 diabetes mellitus as well as a livestock drug. Unlike other drugs, metformin is not metabolized in the body, and almost all of its intake is excreted and released into the aquatic environment via urine and feces, causing adverse effects on aquatic ecosystems. This review provides an overview of the occurrence and detection of metformin in the aquatic environment and its toxic effects on different aquatic organisms (fish, daphnia, rotifers, chlorella). Metformin has been documented in a variety of aqueous environments such as wastewater, surface water, and groundwater as well as drinking water. The wide distribution of metformin in the aqueous environment calls for the development of more accurate detection methods. This paper reviews detection methods for metformin in the aqueous environment and evaluates their advantages and disadvantages. Toxicity studies have shown that metformin can cause adverse reactions in fish, such as oxidative stress, genotoxicity, disruption of intestinal flora, and morphological alterations; it also affects the growth and reproduction of small aquatic organisms. Knowledge gaps in the field of metformin research were assessed, and future research priorities were identified.

## 1. Introduction

The environmental occurrence, distribution, and transport of drugs in aquatic environments have been extensively studied over the years [1]. Drugs in wastewater and receiving water are less dense than other organic chemicals, but their use in human medicine and veterinary medicine is increasing [2]. The use of pharmaceutical products for medical purposes has doubled in recent years, and many high-volume beta blockers, analgesics, antibiotics, lipid modulators, and anti-inflammatory drugs are increasingly found in groundwater, surface water, and wastewater [3,4].

Metformin is one such drug that has been used for more than a century. In 2012, Ahmad discovered the first identified metformin transformation product, a yellow-colored chromophore, by chlorination [5]. Armbruster et al. subsequently determined its structure (C_4_N_5_H_6_Cl) and named it Y, in addition to identifying a colorless chloro-organonitrile transformation product (C_4_N_3_H_6_Cl), C, which is derived from chlorination reactions [6]. Metformin is present in aquatic systems along with many other drugs used to improve human and animal health. Unfortunately, however, it can cause severe endocrine disruption and other adverse effects on aquatic biota [7].

Metformin and its transformation products are often present in high concentrations in the aquatic environment and may exhibit variable toxicity in non-target organisms. Therefore, we evaluated and compared methods for the identification of metformin in water bodies, assessed the sources and distribution of metformin in water bodies, and discussed the toxic effects of metformin and its transformation products on different aquatic organisms (fish, daphnids, rotifers, chlorella) with a view to preparing for subsequent studies.

## 2. Property, Production, and Usage of Metformin

Since it was first synthesized in the 1920s, metformin (C_4_H_11_N_5_) has become a first-line antidiabetic drug because of its excellence and cost-effectiveness in the treatment of type 2 diabetes, which affects 6.1% of the world’s adult population (20–79 years old) [8]. Metformin has a wide range of uses and is an effective drug for preventing cardiovascular disease as well as controlling inflammation, which explains its increasing use [9].

Major global manufacturers of metformin include Wanbury and USV. Major manufacturers in China include Shijiazhuang Puli Pharmaceutical, Keyuan Pharmaceutical, Huarun Shuanghe, and Shouguang Fukang Pharmaceutical. Product types include the categories metformin HCL and metformin DC. Different applications include metformin hydrochloride tablets, metformin hydrochloride extended-release tablets, and others.

The high volume of metformin prescriptions and consumption worldwide is well documented. For example, in China, the consumption of metformin in 2019 was about 786 metric tons [3]. In the United Kingdom, metformin is the ninth most prescribed drug [10]. In the United States, metformin is the fourth most prescribed drug, with prescriptions increasing from 54.5 million in 2006 to 85.7 million in 2019 [11].

## 3. Detection Methods of Metformin in Water

The development of an inexpensive and accurate quantitative characterization method for the detection of persistent compounds in wastewater is one of the most challenging issues facing researchers. Metformin and related products are difficult to separate efficiently from wastewater via chromatography due to their specific properties (high alkalinity and polarity) [12].

Detection methods for metformin (Table 1) include high-performance liquid chromatography (HPLC), electrochemical analysis techniques, spectrophotometry, capillary electrophoresis, thin-layer chromatography (TLC), and liquid chromatography–mass spectrometry (LC–MS).

### 3.1. Liquid Chromatography

High-performance liquid chromatography (HPLC) is an important branch of chromatography, and its basic principle is similar to that of other chromatographic methods (liquid–solid chromatography and liquid –liquid chromatography) [21], whereby a liquid is used as the mobile phase and separation of the components to be measured is achieved by passing the components through a column containing a highly efficient stationary phase, which ultimately enters a detector for analysis. For example, Gedawy [13] achieved simultaneous and efficient analysis of gliclazide and metformin hydrochloride using HPLC, in which the limit of detection (LOD) and limit of quantification (LOQ) of metformin hydrochloride could reach 0.8 μg/mL and 2.45 μg/mL, respectively.

Compared with traditional detection methods, HPLC is characterized by high separation performance and good detection sensitivity, and it is able to complete the analysis of most substances without the limitations of volatility and thermal stability of the analytes. However, HPLC analysis is costly, sensitive to temperature changes during analysis, and more time-consuming to analyze compared to gas chromatography [12].

### 3.2. Electrochemical Analysis Techniques

Electrochemical analysis is a qualitative and quantitative analytical method based on the relationship between electrical parameters such as solution potential, conductance, and current and the target substance. Electrochemical analytical techniques are highly sensitive and accurate, have a wide measuring range, and complete analysis quickly, making them suitable for the measurement of toxic substances [22].

Many studies have applied it to the detection of metformin. Tian and Song [14] utilized the promotion of oxidation currents on the surface of a carbon paste electrode by copper (II) ions for the determination of metformin in drugs. Skrzypek [15] used mercury electrode electroreduction for the determination of metformin. However, these studies involved the determination of metformin at high positive or negative potentials, and common interferences can affect the analytical process. Some heavy metal electrodes such as mercury electrodes are highly toxic and not suitable for long-term work. Reaction intermediates or products generated during the analytical process may contaminate the solid electrodes used and thus affect the reliability of the detection. Therefore, there is still a need to develop new electrode materials to compensate for the shortcomings of the existing metformin detection methods.

### 3.3. Spectrophotometry

Spectrophotometry is an analytical technique for measuring changes in the concentration of substances in solution based on the absorption and transmission properties of light [23]. In a spectrophotometer, a beam of light with a continuous wavelength is broken down into different wavelengths and irradiated into a sample solution, and a fitting curve is established based on the wavelengths and absorption intensities for qualitative and quantitative analysis [24]. Munde [16] showed maximum absorption at 224 and 232 nm for both bulk and tablet formulations with a recovery of about 99% for metformin hydrochloride. Attimarad [17] showed drug solubility and maximum detection sensitivity at 233 nm, with LOD and LOQ of 226 ng/mL and 674.5 ng/mL, respectively, in 0.01 N NaOH solution.

Along with its advantages of simple operation, high efficiency, easy maintenance, and high sensitivity, spectrophotometry has the disadvantage that it is mainly suitable for micro-analysis and is not applicable to the determination of large quantities of substances [25].

### 3.4. Capillary Electrophoresis

Capillary electrophoresis is an analytical technique with a capillary-like separation channel and a high-voltage DC field as the driving force. It is a high-efficiency technology used for partial separation of stereoisomers in the pharmaceutical industry [26]. Ali [18] developed a capillary electrophoresis technique for the determination of metformin with LOD and LOQ of 60 and 100 ng/mL, respectively. Metformin has a 99.1% recovery rate in pharmaceuticals, allowing for rapid and accurate quantitative analysis.

Capillary electrophoresis without the need for a pressure pumping system provides efficient separation of trace volumes of specimen solution by electro-osmotic pumping rather than by mechanical transport of the mobile phase through the stationary phase [27,28]. Electro-osmotic pumping produces a plug flow profile rather than a hydrodynamic profile, so the separation column efficiency of capillary electrochromatography is higher than that of high-performance liquid chromatography. The advantages of capillary electrophoresis are simple operation, low sample volume, high separation efficiency, low cost, high separation capacity, high separation speed, and small feed volume. The disadvantages of capillary electrophoresis are that it is less reproducible than HPLC in terms of migration time, injection accuracy, and detection sensitivity [18,29,30].

### 3.5. Thin-Layer Chromatography

Thin-layer chromatography (TLC) is a chromatographic technique in which a stationary phase adsorbent is uniformly applied to a glass plate, and separation is achieved by dissolution and adsorption of the sample components between the stationary and mobile phases. It is commonly used for the identification of drugs and the determination of their contents [31]. Kale [19] proposed a simple, rapid, and accurate high-performance TLC for the estimation of metformin hydrochloride in tablets on silica gel 60 F254 plates using butanol:water:acetic acid as mobile phase. The resolution of the device was as high as 0.35, and the LOD and LOQ for quantification of metformin hydrochloride at 240 nm were 6160.85 ng per band and 18,669.26 ng per band, respectively.

TLC has many advantages, including ease of operation, simple equipment, and easy color development [32]. It has a fast unfolding rate, usually only 15–20 min; separating the mixture is not difficult, and the resolution is usually 10–100 times higher than that of paper chromatography. TLC has a wide range of applications: it can be used for both micro-sample separation and large-sample preparation and can be used with corrosive colorants such as concentrated sulfuric acid and hydrochloric acid. It is also relatively inexpensive, and one plate can be used for the simultaneous detection of multiple samples [33,34,35].

The disadvantage of TLC over HPLC is its poor separation of biomolecules [36].

### 3.6. Liquid Chromatography–Mass Spectrometry

Liquid chromatography–mass spectrometry (LC–MS) combines liquid chromatography technology with mass spectrometry. The working principle is to separate the samples by liquid chromatography and then detect and analyze them by mass spectrometry, so as to obtain the qualitative, quantitative, and structural analysis of the samples [37].

Al Bratty [20] used LC–MS for the determination of metformin and other gliptins in human plasma according to the International Conference on Harmonization/FDA guidelines. The study was carried out on a Chromolith high-resolution RP-18e HPLC column using ammonium formate buffer: acetonitrile for isocratic elution. The limit of detection was 17.8 ng/L.

LC–MS has high sensitivity and specificity, more accurate identification of compound structure, and reliable quantitative and qualitative results. However, its disadvantage is that it is expensive and has high maintenance costs [38,39].

In conclusion, for the analysis of metformin, HPLC and LC–MS are recommended due to their feasibility, accuracy, and other advantages. HPLC is more economical because of the high costs of the LC–MS instrumentation and its maintenance.

## 4. Sources and Distribution of Metformin in Water Bodies

Unlike most medications, metformin is not metabolized in the body; almost all of the amount taken is excreted in the urine and feces [40]. As a result, the widespread use of metformin since 1999 has led to frequent detection of metformin in aquatic systems globally. The occurrence of metformin in the environment is attributed to its lack of metabolism in living organisms and the limited effectiveness of treatment processes in existing wastewater treatment plants.

### 4.1. Sources of Metformin in Water Bodies

**Figure 1:**
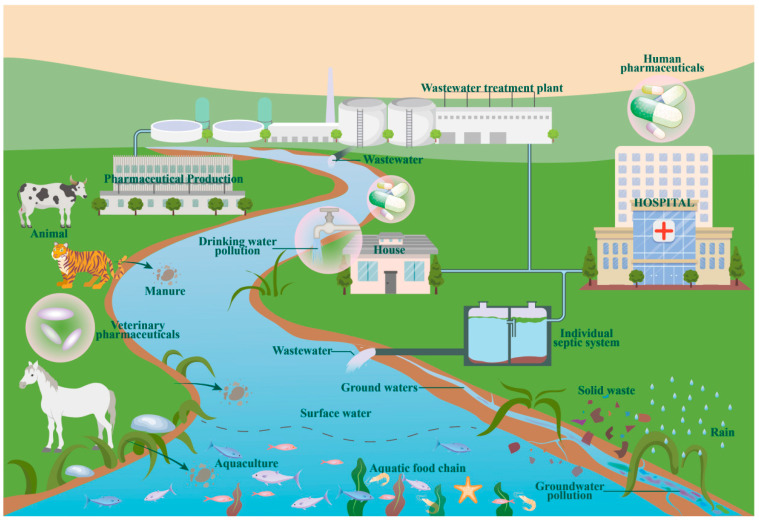
Sources of metformin in water bodies.

Metformin is one of the most commonly found drugs in the aquatic environment. Metformin is present due to various medical prescriptions for managing chronic diseases (e.g., type 2 diabetes, polycystic ovary syndrome, and certain cancer treatments). Unlike other drugs, metformin is excreted intact in nature [41,42,43]. As a common prescription drug, it is mainly derived from manufacturing in pharmaceutical factories, used by humans and animals, and discharged into the environment. Thus, the sources of metformin in water bodies can be categorized into three groups: human medication, livestock medication, and pharmaceutical factory discharges (Figure 1) [44].

Because metformin is a first-line antidiabetic drug, a large number of prescriptions are issued from hospitals for the treatment of various related diseases. The use of these drugs is mainly divided into autonomous use at home and use in hospital wards, where solid waste and wastewater containing metformin are generated through methods such as excretion and unintentional disposal. When the solid waste comes into contact with surface water, the metformin moves from the solid waste into the water body [45]. The wastewater generated such as urine, domestic sewage, etc., comes to the water bodies through wastewater treatment plants and septic tanks.

Metformin is also a common veterinary drug, used in livestock farming and aquaculture. The drug makes its way from animals into bodies of water via two paths: one is through the excretion of feces into water bodies, and the other is via surface runoff from rainfall on a decomposing carcass [46].

Pharmaceutical factories also produce a large amount of wastewater containing metformin during the drug production process. The sewage or wastewater treatment plant cannot completely degrade metformin, so metformin enters water bodies with the plant discharge, resulting in pollution [47].

### 4.2. Metformin Distribution in Water Bodies

Metformin has a low octanol–water partition coefficient (log *K*ow, −2.64) and is readily soluble in water and difficult to retain or deposit in soil [48]. Therefore, metformin levels in environmental water have been found to increase over time as the diabetic population and the associated prescription medications increase.

Metformin is widely present in a variety of aqueous environments, such as wastewater, surface water, rainwater, groundwater, and drinking water matrices, and has been detected in aquatic systems in 91 countries at levels ranging from 0.2 ng/L to 1180 μg/L, spanning seven orders of magnitude. In the United States, the maximum concentration of metformin in the influent of wastewater treatment plants was 36,100–73,300 ng/L, and the maximum concentration of metformin in the effluent was 338–30,700 ng/L, with a treatment rate of 73.6–96.5% [40]. It is noteworthy that metformin values in the wastewater from the WWTP remained high, probably because the wastewater was treated with a biological system, which was inefficient in removing metformin [49]. Tisler and Zwiener’s study [40] showed that in one of the wastewater treatment plants, the GUA concentration was higher than the metformin concentration, but the two did not show a reciprocal transformation relationship, suggesting the possibility of the existence of other transformation pathways of metformin, such as MBG, 2,4-DAT, and 2,4-AMT. In this study, the concentration of MBG-1 was detected in the effluent at 0.122 μg/L. The effluent’s 2,4-DAT and 2,4-AMT concentrations were higher than those of the influent water, showing a similar increasing response trend.

There is growing evidence that metformin and its transformation products are formed through or in sewage treatment plants, implying that sewage is a major source of these pollutants in surface waters [50]. The accumulation of metformin and its transformation products in the environment is related to the amount of use, the effectiveness of sewage treatment plant purification, and the dilution factor of surface water [51]. Consequently, concentrations of metformin and its transformation products in receiving waters are expected to reach 3–4308 ng/L in surface water. Groundwater resources are also at serious risk from metformin and its metabolites. The highest concentration of metformin in 17 domestic wells in northern China reached 0.045 μg/L, with a detection frequency of 7.4% [52]. The presence of metformin was also frequently detected in groundwater in the Mezquital Valley, which serves as a wastewater treatment center for Mexican urban metropolis, where the highest concentration reached 0.029 μg/L [53]. In US drinking water, metformin was found up to 30, 33, 60–65 ng/L [40].

## 5. Toxic Effects of Metformin on Aquatic Organisms

The occurrence of metformin and its transformation products in aquatic environments has become an increasingly important issue due to their metabolic and water-soluble properties. There is much evidence that these contaminants can have adverse effects on non-target organisms through endocrine disruption. The ecological risks posed by metformin and its transformation products are increasing in the context of prolonged and heavy use and may become a global threat.

### 5.1. Mode of Entry into Organisms and Enrichment

The bioabsorption, in vivo distribution, and excretion of metformin are closely related to its biotransport and metabolic transformation, as well as to its in vivo enrichment, accumulation, and toxic effects [54]. The ways in which environmental pollutants enter organisms can be divided into two categories: passive transport and specialized transport. Passive transport biofilms do not play an active role in the transport of substances, and specialized transport biofilms do [55]. Since metformin is not easily degraded in the body, metformin that enters the body is eliminated through excretion or is enriched in the body.

A study on *Typha latifolia* [56] showed that plants take up metformin from the external environment through the root system and transfer it to other tissues such as rhizomes and leaves. Ussery et al. [57] exposed embryonic and larval stages of Japanese medaka fish to 10 μgL^−1^ of metformin for 24 or 168 h. Their results suggest that the hardening of the chorioallantoic membrane of metformin affects the uptake and accumulation of metformin. They determined the amount of metformin in larvae and observed the ability of medaka fish larvae to remove metformin from their bodies. Medaka fish larvae showed strong environmental adaptation, reaching a maximum concentration of 3120 μg/g of metformin after exposure, but the amount of metformin was reduced below the limit of detection within 24 h after removal from the exposure environment. The body load of metformin in the larvae of medaka fish was determined by the results of a study of the body load of metformin in the larvae of medaka fish.

### 5.2. Toxic Effects

#### 5.2.1. Toxic Effects of Metformin in Fish

The antidiabetic drug metformin is one of the pharmaceutical compounds deposited in large quantities as a result of human activities, and its global presence in hydrological basins and municipal wastewater treatment plants is of great concern and adversely affects aquatic biota, including fish (Table 2).

Metformin has a delayed senescence effect in fish. Delayed accumulation of lipofuscin in the liver, aging-associated β-galactosidase (SA-β-gal) activity in the skin, and a significant reduction in serum cholesterol and triglyceride levels have been found in 10-month-old fish, as exemplified by *Nothobranchius guentheri* [58,59]. Behavioral testing found improved motor, learning, and memory skills with decreased SA-β-gal activity and neural progenitor fiber degeneration, suppressed inflammatory responses including downregulation of NF-κB and pro-inflammatory cytokine expression, and enhanced anti-inflammatory cytokine levels [58]. These findings suggest that metformin extends lifespan and exerts neuroprotective and anti-inflammatory functions to improve cognitive performance in annual fish (fish with an average lifespan of 12 months). Metformin activation of AMPK and SIRT6 reduces NF-κB-mediated inflammation and resists oxidative stress by enhancing FoxO1a and PGC-3α expression, ultimately delaying senescence-like phenomena in vertebrate intestines as evidenced by a reduction in the activity of the senescence biomarker SA-β-gal [59].

Nielsen et al. showed that metformin exposure differentially affected growth and morphology in two cohorts of *Pimephales promelas*. Adverse outcomes of malformations, including skeletal abnormalities, cardiac edema, yolk sac edema, and ocular abnormalities, were observed in the experiment, suggesting disturbances in energetic homeostasis and visual effects, and that adaptations of wild spawning larvae were more affected than those of laboratory spawning fish [60].

Metformin also causes oxidative stress. A study on *Labeo rohita* found that 40 μg/L and 80 μg/L of metformin led to an increase in reactive oxygen species in fish, which in turn caused the development of oxidative stress while interfering with nonspecific immune responses. In addition, animals in the highest dose group (80 μg/L) exhibited severe DNA damage, implying that metformin is quite genotoxic [63]. Studies on *Clarias gariepinus* have shown that high doses (50 mg/L) of metformin induce oxidative stress in cells by reducing superoxide dismutase (SOD) antioxidant enzymes and total antioxidant capacity (TAC) [64].

Metformin has been shown to cause gut microflora disruption. Larval microbiomes of *Pimephales promelas* were dominated by the ascomycetes and thick-walled ascomycetes, with a slight increase in ascomycetes and a decrease in thick-walled ascomycetes as metformin exposure increased [61]. Similar results were found in a study on *Salmo trutta f. fario* [65].

Metformin exposure in *Astyanax lacustris* resulted in plate fusion, capillary dilatation hyperplasia, and loss of microridges; these observed morphological changes may interfere with gill physiology [66]. In *Pimephales promelas*, the onset of reproduction was delayed by 9–10 d in the metformin medium-concentration (31 μg/L) and high-concentration (322 μg/L) treatments [62]. In *Oryzias latipes*, 6 metabolite alterations, 66 protein alterations, and 2 gene alterations were found after exposure to 100, 32, 10, 3.2, and 1.0 ng /L metformin [67]. In summary, metabolomics, proteomics, and gene expression data suggest that metformin is able to affect the overall health of early life-stage (ELS) fish by disrupting biomolecular metabolism, cellular energy, and neural development. Additionally, a long-term and short-term exposure experiment showed that metformin interferes with motor behavior in zebrafish. This is consistent with the results of differential expression of some genes related to neurodevelopment after metformin exposure [68].

In summary, metformin causes a variety of adverse effects in fish, posing a serious threat to the growth, development, and reproduction of these important aquatic organisms. These studies are a reminder of the endocrine-disrupting effects of metformin. Even at very low concentrations, chronic exposure is capable of disrupting secretion-disrupting homeostasis in fish, which in turn affects reproduction and impairs population levels.

#### 5.2.2. Effects of Metformin on Other Test Organisms

##### Daphnia

Acute (48 h) and chronic (14 d) toxicity tests were conducted on *Daphnia similis*. In the acute test, immobilization of *Daphnia similis* was observed, while in the chronic exposure test, reproduction of *Daphnia similis* was disturbed. In the acute exposure experiment, its EC_50_ was 14.3 mg/L; in the chronic exposure experiment, its EC_10_ (corresponding to the non-observed effect concentration, NOEC) was 4.4 mg/L [69].

##### Rotifers

*B. calyciflorus* and *P. patulus* were cultured separately starting from single individuals. Experiments were conducted using 25 mL of test medium and five concentrations of 0, 25, 50, 100, and 200 μg/ L for exposure. The number of rotifers living in each jar was quantified daily, and then the surviving individuals were transferred to fresh jars containing the appropriate metformin–algae combination. The experiment was terminated after 16 days. The results showed that metformin adversely affected the population abundance and population growth rate (r) of both rotifer species. The results of the study showed that the possible maximum acceptable toxicant concentration (LOEC) for *B. calyciflorus* and *P. patulus* were 50 μg/L and 25 μg/L, respectively [70].

##### Chlorella

Freshwater *Chlorella vulgaris* cultures were exposed to different concentrations of metformin (0–767.9 mg L^−1^) to verify whether metformin disrupts the conduct of photosynthesis and to explore its molecular mechanism. The results showed that metformin was able to impair photosynthesis in *Chlorella vulgaris* through dual regulation, including inhibition of the downstream action of electron transport chain complex I and activation of the expression of SnRK1 (sucrose non-fermentation-associated kinase 1). The LOEC of fish may be between 1.5 mg/L and 76.8 mg/L [71].

## 6. Outlook

Available data indicate that metformin and its metabolites are widely present in the aquatic environments of sewage, surface water, groundwater, and drinking water, with sewage being the main source of these pollutants in surface water. Therefore, special attention should be paid to the ecological risk of metformin in areas such as wastewater treatment plants. Currently, few studies have focused on the transport processes of metformin in different environmental compartments, which is crucial for understanding the environmental fate of metformin, and more attention needs to be devoted to this.

This review summarizes the existing metformin detection methods, among which HPLC and LC–MS can detect and quantify metformin most accurately and efficiently. Of the two, HPLC is more economical.

This paper also reviews the toxic effects of metformin and its metabolites on different aquatic organisms. The data suggest that metformin can cause adverse effects through different modes of action (oxidative stress, genotoxicity, disruption of intestinal flora, morphological alterations, etc.). However, these studies were mainly acute exposure experiments, where exposure concentrations were usually significantly higher than environmental concentrations, and could not effectively account for ecological risks in the environment, so further studies should focus on chronic toxicity at environmental concentrations.

More toxicological data from different species—including some small aquatic organisms, as they are the basic components of aquatic ecosystems—are needed to better understand the ecological risks of metformin. In addition, there is a lack of information on metformin metabolites, so future studies on the identification of metformin metabolites and their toxic effects are necessary.

## Figures and Tables

**Table 1:** Comparison of detection methods for metformin in water.

Detection Methods	Advantages	Limitations	Applicability	LOD	LOQ	References
High-performance liquid chromatography (HPLC)	High separation performance and good detection sensitivity; without the limitations of volatility and thermal stability of the analytes	Costly, sensitive to temperature changes, more time-consuming than gas chromatography	Metformin hydrochloride	0.8 mg/L	2.45 mg/L	[13]
Electrochemical analysis techniques	Highly sensitive and accurate, have a wide measuring range, and complete analysis quickly	Suffer from interference, heavy metal electrodes are toxic and not suitable for long-term work, reaction intermediates or products influence the results	Metformin	-	-	[14,15]
Spectrophotometry	Simple operation, high efficiency, easy maintenance, and high sensitivity	Not suitable for the determination of large quantities of substances	Metformin	226 mg/L	674.5 mg/L	[16,17]
Capillary electrophoresis	Simple operation, low sample volume, high separation efficiency, low cost, high separation capacity, high separation speed, and small feed volume	Less reproducible than HPLC in terms of migration time, injection precision, and detection sensitivity	Metformin	60 mg/L	100 mg/L	[18]
Thin-layer chromatography (TLC)	Ease of operation, simple equipment, and easy color development, Wide range of applications, Shorter time, higher resolution than paper chromatography, relatively cheap	Poor separation of biomolecules compared to HPLC	Metformin hydrochloride	6160.85 ng per band	18,669.26 ng per band	[19]
Liquid chromatography–mass spectrometry (LC–MS)	High sensitivity and specificity, more accurate identification of compound structure, and reliable quantitative and qualitative results	High instrumentation and maintenance costs	Metformin	-	17.8 ng/L	[20]

**Table 2:** Toxic effects of metformin on fish.

Fish	Concentration	Negative Impact	References
*Nothobranchius guentheri*	2 mg/g (food)	-Increased longevity -Improvement of cognitive skills -Suppression of the inflammatory response	[58]
	2 mg/g (food)	-Delayed aging -Resistance to oxidative stress and inflammation	[59]
*Pimephales promelas*	5 and 50 μg/L	-Energy homeostasis disorders and visual effects	[60]
	0.02, 3.44, 33.6, 269 μg/L	-microbial flora disorder	[61]
	3.0, 31, 322 μg/L	-Delayed onset of reproduction 9–10 d	[62]
*Labeo rohita*	40 and 80 μg/L	-Increased reactive oxygen species and free radical production, DNA damage	[63]
*Clarias gariepinus*	10 and 50 mg/L	-Suppression of immunity in exposed treated fish can be through activation of lymphocytes and monocytes -Induces cellular oxidative stress by reducing superoxide dismutase (SOD) antioxidant enzymes and total antioxidant capacity (TAC) -Increased expression of the inflammatory mediatorsinterleukin-6 (IL-6) and interleukin-1β (IL-1β)	[64]
*Salmo trutta f. fario*	1, 10, 100, and 1000 μg/L	-In vitro expression of fish pathogen virulence genes induced by metformin and the effect of metformin on microbiome composition in juvenile brown trout (Oncorhynchus mykiss)	[65]
*Astyanax lacustris*	50, 100, 1000, and 10,000 μg/L	-Plate fusion, capillary dilatation and proliferation, and disappearance of microridges, observed morphological changes that may interfere with gill physiology	[66]
*Oryzias latipes*	1, 3.2, 10, 32, and 100 ng/L	-Alteration of many important pathways associated with the overall health of ELS fish, including biomolecular metabolism, cellular energetics, nervous system function/development, cellular communication and structure, and reactive oxygen species detoxification	[67]
*Danio rerio*	1, 10, 100, 1000, and 10,000 ng/L	-Motor activity was affected and several genes involved in neurological and cardiovascular development were differently expressed after exposure to metformin.	[68]

## Data Availability

Not applicable.
